# Broadened Population-Level Frequency Tuning in Human Auditory Cortex of Portable Music Player Users

**DOI:** 10.1371/journal.pone.0017022

**Published:** 2011-03-02

**Authors:** Hidehiko Okamoto, Henning Teismann, Ryusuke Kakigi, Christo Pantev

**Affiliations:** 1 Institute for Biomagnetism and Biosignalanalysis, Westfalian Wilhelms-University Muenster, Muenster, Germany; 2 Department of Integrative Physiology, National Institute for Physiological Sciences, Okazaki, Japan; Hotchkiss Brain Institute, University of Calgary, Canada

## Abstract

Nowadays, many people use portable players to enrich their daily life with enjoyable music. However, in noisy environments, the player volume is often set to extremely high levels in order to drown out the intense ambient noise and satisfy the appetite for music. Extensive and inappropriate usage of portable music players might cause subtle damages in the auditory system, which are not behaviorally detectable in an early stage of the hearing impairment progress. Here, by means of magnetoencephalography, we objectively examined detrimental effects of portable music player misusage on the population-level frequency tuning in the human auditory cortex. We compared two groups of young people: one group had listened to music with portable music players intensively for a long period of time, while the other group had not. Both groups performed equally and normally in standard audiological examinations (pure tone audiogram, speech test, and hearing-in-noise test). However, the objective magnetoencephalographic data demonstrated that the population-level frequency tuning in the auditory cortex of the portable music player users was significantly broadened compared to the non-users, when attention was distracted from the auditory modality; this group difference vanished when attention was directed to the auditory modality. Our conclusion is that extensive and inadequate usage of portable music players could cause subtle damages, which standard behavioral audiometric measures fail to detect in an early stage. However, these damages could lead to future irreversible hearing disorders, which would have a huge negative impact on the quality of life of those affected, and the society as a whole.

## Introduction

It is well-established that exposure to loud noise can cause hearing impairment, hyperacusis, and tinnitus [1], [2], [3], [4], [5], [6], [7]. The detrimental effects of noise exposure have so far mainly been discussed with respect to occupational environments [8]; however, the research focus of neuroscience has recently shifted towards the role of recreational sounds like music, which have a continuously increasing impact on human life [9], [10]. Currently, so-called portable music players (PMP) with insert earphones are regularly used by numerous people [11], particularly by adolescents and young adults, and very often in noisy environments like trains or busses. In such environments, many users tend to listen at very high volumes to overcome the intense surrounding noise [12]. Even though it appears likely that exposure to very loud music via PMPs would lead to damage in the hearing system, this matter is still debated [13], [14]. There are studies [15], [16], [17], [18], however, that demonstrated that daily PMP usage can lead to hearing impairment and tinnitus, both of which are among the most common diseases in industrialized countries [3], [19].

In most studies on PMP usage, the detrimental effects on the hearing system have been evaluated in terms of behavioral and subjective outcome measures (e.g. hearing threshold, speech test, etc.). However, PMP misusage may subtly damage the hearing system already after a few years of malpractice, and conventional behavioral measures may not be sensitive enough to detect these subtle damages, because it might take some time until they impair performance or become noticeably disabling. Non-invasive electrophysiological methods like magnetoencephalography (MEG) are useful alternatives, because they can objectively measure auditory brain activity even in distracted listening and might be capable of detecting sound exposure-related malfunctions of the brain before these become behaviorally measurable.

**Figure 1:**
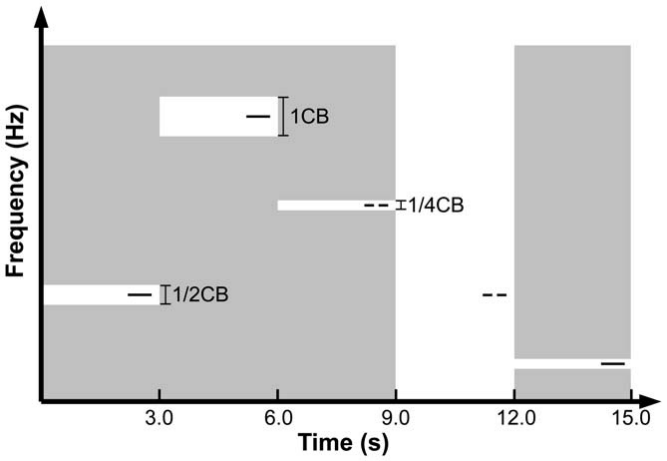
Experimental design. The figure illustrates the time course (x-axis) of the stimulation, and the frequency spectra (y-axis) of the test stimuli (TS) and the band-eliminated noises (BEN). Black lines represent pure tone TS. The target TS contained a temporal gap of 0.01 sec duration, which subjects were supposed to detect and to indicate via button press during the focused listening condition. The gray area represents energy of the noise; white areas represent noise energy absence due to eliminated bands or lack of BEN stimulation. The width of the eliminated band in the BEN (white areas) was either 1/4, 1/2, or 1 critical band (CB). The eliminated band was strictly centered at the TS frequency, which was either 250, 350, 450, 570, 700, 840, 1000, 1170, 1370, 1600, 1850, 2150, 2500, 2900, 3400, or 4000 Hz.

**Figure 2:**
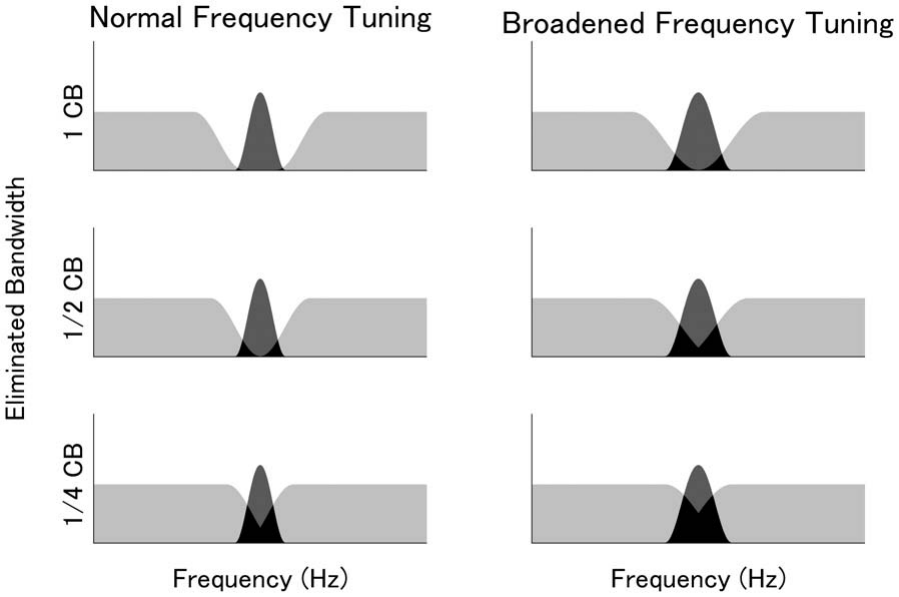
Models of population-level frequency tuning. Left and right columns represent schematic illustrations of normal and broadened population-level frequency tuning. The three differently colored areas represent, (i) neural activity elicited merely by band-eliminated noises (BEN: light gray areas), (ii) neural activity elicited merely by test pure tones (TS: dark gray areas), and (iii) neural activity elicited by both BEN and TS (black areas). The rows represent wide (top: 1 critical band (CB)), middle (medium: 1/2 CB), and narrow (bottom: 1/4 CB) BEN conditions. The dark gray areas represent the N1m response amplitude as elicited by the TS-onset; the neural activity represented by the black areas has been masked by the simultaneously presented (but earlier on-setting) BEN. Notably, the dark gray areas (i.e., N1m amplitudes evoked by the TS) are less influenced by the eliminated bandwidths in case of normal frequency tuning compared to broadened frequency tuning, especially in the narrow BEN condition.

Previous studies [20], [21], [22], [23], [24] have established a method to objectively measure population-level frequency tuning in human auditory cortex by using tonal test stimuli (TS), which are presented either in isolation or embedded in band eliminated noise (BEN)(c.f. Figure 1). The neurons of the auditory cortex activated by TS or BEN overlap partially, and the degree of overlap depends on both the width of the eliminated band of the BEN and the sharpness of the population-level frequency tuning (Figure 2). Wide BENs and sharp tuning (in contrast to narrow BENs and broadened tuning) result in sparse overlap, leading to large neural activity amplitude elicited by the TS onset. Moreover, the effect of sharp tuning on evoked brain activity amplitude is stronger in case of narrow BENs, which is reflected in a rather small TS-evoked activity amplitude difference between wide and narrow BEN conditions [20], [22], [23]. Here, by means of MEG, we objectively measured the auditory evoked brain responses of two groups of young subjects (PMP “exposed” group and “control” group) to examine the hypothesis that daily and inappropriate PMP usage could broaden the frequency tuning *before* malfunctions become noticeable and detectable with standard behavioral audiometric examinations.

## Results

### Behavioral Performance

Hearing thresholds for pure tones ranging from 125 Hz to 8000 Hz, speech test, and hearing-in-noise test [25] results did not significantly differ between the “exposed” and “control” groups. The behavioral responses in each band-eliminated noise (BEN) condition during the MEG sessions were also similar between the two groups with regard to reaction time (Table 1) and error rate (Table 2). Repeated-measures analyses of variance (ANOVA) resulted in significant main effects for BEN-TYPE (reaction time: F (3, 72) = 17.6, p<0.001; error rate: F (3, 72) = 183.1, p<0.001), but neither significant main effects for GROUP nor significant interactions between BEN-TYPE and GROUP were observed. Therefore, the behavioral results indicate that the participants performed better in case of wider BEN conditions. However, there was no significant behavioral difference between the two groups in the focused listening condition.

**Table 1:** Mean (±SD) reaction time (sec) for each band-eliminated noise (BEN) condition.

	Reaction Time (sec)			
Groups:	BEN_1/4CB	BEN_1/2CB	BEN_1CB	No BEN
Exposed	0.417±0.089	0.403±0.069	0.394±0.074	0.362±0.079
Control	0.459±0.088	0.443±0.092	0.431±0.095	0.393±0.083

**Table 2:** Mean (±SD) error rate (%) for each band-eliminated noise (BEN) condition.

	Error Rate (%)			
Groups:	BEN_1/4CB	BEN_1/2CB	BEN_1CB	No BEN
Exposed	17.1±5.9	12.8±5.8	6.0±7.2	2.9±3.5
Control	17.0±4.7	13.7±4.3	6.1±4.2	3.5±4.0

### Auditory Evoked Fields

**Figure 3:**
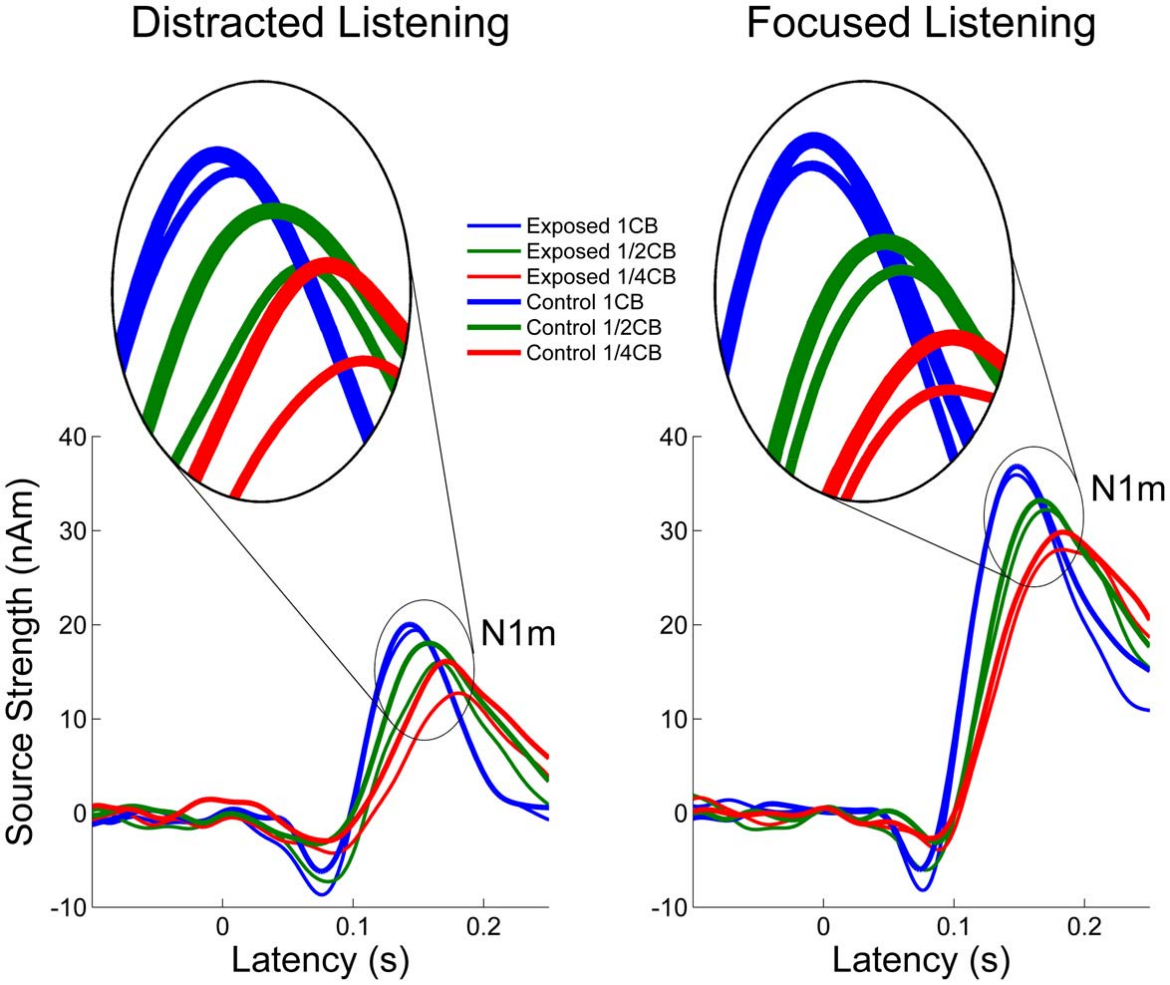
Source strength waveforms. Grand averaged source strength waveforms for each experimental condition. Left and right panels represent the distracted and focused listening conditions, respectively. Thin and thick lines represent the portable music player (PMP) exposed and the control groups. Color codes band-eliminated noise (BEN) condition (blue  =  1 critical band (CB), green  =  1/2 CB, red  =  1/4 CB). All source strength waveforms depicted clear N1m responses, which are further magnified in the ellipsoids. N1m source strengths were overall larger in the focused listening than in the distracted listening condition.

The iso-contour field maps of the N1m responses elicited by the pure tone test stimuli (TS) showed clear dipolar patterns over both hemispheres, confirming that the use of the single dipole approach was appropriate. The goodness-of-fit of the obtained equivalent current dipole model was not significantly different between groups and sessions (mean ± SD: exposed - distracted 95.5±3.02%, control - distracted 95.5±3.07%, exposed - focused 94.1±3.07%, control - focused 95.8±2.96%). The N1m responses in the distracted listening condition and in the narrow BEN condition were delayed and reduced in amplitude as compared to the focused listening condition and the wide BEN condition (Figure 3). The source strength waveforms were similar between exposed and control groups in the focused listening condition; however, they were different during distracted listening, especially in the BEN_1/4CB condition.

**Figure 4:**
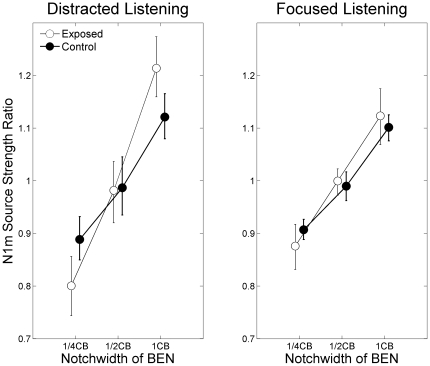
Normalized N1m source strength. The graphs display the group means (N = 13) of the normalized N1m source strengths for each band-eliminated noise (BEN) condition during distracted (left panel) and focused (right panel) listening. Open and filled circles represent the exposed and control groups with the error bars denoting the 95% confidence limits.

Figure 4 represents the mean N1m source strength ratio in each condition and in each subject group. The repeated-measures ANOVA in the distracted listening condition resulted in a significant main effect for BEN-TYPE (F (2, 50) = 46.1, p<0.001), and a significant interaction between GROUP and BEN-TYPE (F (2, 100) = 3.7, p<0.03). In general, narrower BENs caused smaller N1m source strength ratios in both groups; however, this effect was more obvious in case of the exposed compared to the control group. In contrast, for the focused listening condition the repeated-measures ANOVA showed only a significant main effect for BEN-TYPE (F (2, 50) = 50.7, p<0.001), but no significant interaction between GROUP and BEN-TYPE (F (2, 100) = 0.7, p = 0.48).

## Discussion

The present study experimentally confirmed the hypothesis that the extensive usage of PMPs could have negative impacts on the hearing system. The objective neurophysiological MEG results showed a significant interaction between GROUP and BEN-TYPE during distracted listening, suggesting that the population-level frequency tuning was significantly broadened in the “exposed” group compared to the “control” group (c.f. Figure 2) [20], [22], [23]. However, there were no significant differences in the behavioural measures (pure tone audiogram, speech test, hearing-in-noise test [25], and the button press responses during the MEG measurements (Tables 1 and 2)), which can only be obtained under focused listening conditions. Additionally, the MEG results were similar between groups in the focused listening condition. Therefore, the obtained results suggest that PMP usage can cause subtle damages, which at the moment can easily be compensated by focused attention in the hearing system, and which therefore cannot be detected by standard behavioral measures.

Sensory neural networks are composed not only of excitatory neurons, but also of inhibitory neurons, which enable the suppression of irrelevant neural activity. Both lateral inhibition [22], [26], [27], [28], [29], [30] and co-tuned [31], [32] neural mechanisms can sharpen the auditory frequency tuning by suppressing excitatory activity of neighboring neurons, leading to improved spectral contrast, and resulting in improved auditory performance. Damage to inhibitory neural networks may not worsen the hearing threshold, but may broaden the usually sharp frequency tuning of the auditory system. Therefore, the integrity of the inhibitory system may not mainly influence the threshold of excitatory neurons and thus the perceptual sensitivity of the auditory system per se, but it may play an important role for the *quality* of auditory perception [26], [33]. However, the inhibitory connections along the auditory pathway have been demonstrated to be particularly sensitive to damages by noise exposure [34], [35], [36], [37], [38].

The broadened auditory frequency tuning in the exposed compared to the control group for the distracted listening condition might reflect beginning damage of the auditory pathway due to regular exposure to loud music via PMP. For instance, Calford et al. [34] reported that neurons in cat primary auditory cortex exhibited expanded response areas following exposure to intense pure tones. This finding can be explained in terms of the loss of inhibition [39]; the intense pure tones preferably damage inhibitory neurons, which contribute to the sharpening of frequency tuning in auditory cortex [33]. Noteworthy, loss of inhibition in the auditory pathway has also been argued to be closely related to the generation and maintenance of tinnitus [40]. Our present results indicated broadened population-level frequency tuning expressed in the auditory cortex of the exposed subjects during distracted listening; however, it is not possible to conclude with certainty from the present results where exactly in the auditory pathway the broadening of the frequency tuning has its origin.

One might argue that the “exposed” subjects were better trained to ignore unwanted auditory signals by their daily PMP usage, and that they thus could better suppress brain activity corresponding to the auditory signals during distracted listening compared to the “control” subjects. If this was the case, the “exposed” subjects should have exhibited equally reduced N1m responses in all BEN conditions. However, in the distracted listening condition there was no significant main effect of GROUP, but a significant interaction between GROUP and BEN-TYPE (Figures 3 and 4). Therefore, it appears unlikely that the “exposed” subjects could better filter out sound signals compared to the “control” ones.

The MEG results of the attentive listening session are in line with the behavioral data, which did not indicate performance differences between groups. We interpret this finding as indication that the detrimental effects of the PMP misuse, even though already objectively measurable in the auditory cortex during passive listening, were not yet noticeable and behaviorally relevant, probably because they still could be compensated by focusing attention. This interpretation is supported by the finding that focused attention can sharpen population-level frequency tuning [20], [22], [23]. However, the broadened frequency tuning observed in the PMP exposed subjects could already compromise everyday performance under certain distracted conditions – in the worst case, a distracted person with broadened frequency tuning might miss another person's warning call regarding an approaching car in a noisy traffic situation. Moreover, the broadened frequency tuning might be a precursor to forthcoming symptoms. Even though millions of PMP exposed people might not yet have observable symptoms, the attentional compensation mechanisms will likely break down in the medium or long term. Continuous PMP misuse can cumulatively damage the hearing system and lead to permanent hearing impairment and tinnitus.

### Conclusions

Here, we suggested that the population-level frequency tuning was broadened in young subjects who had regularly and extensively used portable music players. The present findings indicate the necessity of thorough education regarding appropriate portable music player usage for adolescents and young adults. It is a major health policy issue to prevent permanent hearing impairment and chronic tinnitus in increasingly larger numbers of portable music player users.

## Materials and Methods

### Subjects

We compared two experimental groups. The “exposed” group consisted of 13 young subjects who had listened to music via PMP regularly (mean ± SD: 1.86±0.79 hours per day) for at least the last two years. Their average comfortable volume setting for pop music (as measured in an acoustically shielded room) was 80.9 (±10.2) dBA sound pressure level. Thus, it appears very likely that they would listen with even larger and closer to harmful volumes in noisy environments. The “control” group consisted of 13 young subjects who had not yet used PMPs regularly. The two groups were matched with regard to age (exposed: 23.0±4.4 years; control: 24.8±3.3 years) and gender (10 females, 3 males). All subjects gave written informed consent for their participation. The study was performed in accordance with the Declaration of Helsinki, and the ethics committee of the Medical Faculty, University of Muenster approved the study.

### Experimental Design

We measured the auditory evoked fields elicited by tonal test stimuli (TS), which are presented either in isolation or embedded in band eliminated noise (BEN) (Figure 1). The TS (0.6 sec duration with 0.01 sec on- and off-set ramps) were pure tones with frequencies of either 250, 350, 450, 570, 700, 840, 1000, 1170, 1370, 1600, 1850, 2150, 2500, 2900, 3400, or 4000 Hz (one critical band (CB) steps [41]). The TS frequencies were presented in a pseudo-randomized manner in order to avoid involuntary bottom-up driven sequencing effects on the auditory evoked fields [21]. The simultaneously presented BENs were broadband noises containing eliminated bands with widths of either 1/4, 1/2, or 1 CB centered at the embedded TS frequency (Figure 1). The width of the CB is closely related to the width of the auditory filter [41]. Masking effects of noise on pure tones are mainly caused by spectral components of the noise invading the critical bands of the tones. Therefore, BENs with narrower eliminated bands can cause larger masking effects and result in smaller auditory evoked responses elicited by the test pure tones than BENs with wider eliminated bands. The behavioural and MEG data were analyzed after pooling across all TS frequencies, resulting in 192 epochs for each BEN condition. Half of the TS contained a silent gap of 0.01 sec duration (with 0.01 sec rise and fall times) in the middle of the TS, and the other half did not. All TS and BENs were presented binaurally. The hearing threshold for the 1000 Hz TS was determined for each ear individually before the MEG measurement. The 1000 Hz TS was presented with intensity of 38 dB above individual sensation threshold. The other TS were presented with identical power as the 1000 Hz TS. The BENs were presented with 12 dB larger total power than the TS.

Given the evidence that auditory focused attention can improve the population-level frequency tuning in human auditory cortex [20], [22], [23], we measured auditory evoked fields under two attentional conditions (distracted vs. focused listening). In the first MEG session, the participants were measured under distracted listening conditions – here, they were instructed to attend to a silent movie and to memorize its content. Compliance was assessed afterwards by asking the subjects content-related questions. The second MEG session was performed under focused listening conditions. In this case, the participants were instructed to press a response button as quickly as possible with their right index finger when they detected a temporal gap within the auditory TS. Auditory evoked fields were measured with a whole-head gradiometer system (275 channels, Omega; CTF Systems, Coquitlam, British Columbia, Canada) in a silent magnetically shielded room. The auditory evoked fields elicited by the TS (irrespectively of both TS frequency and presence of a temporal gap) were selectively averaged for each BEN condition (BEN_1/4CB, BEN_1/2CB, BEN_1CB and no BEN) after rejection of artefact epochs containing large field changes (>3 pT). For the analysis of the N1m response [42], the averaged magnetic fields were low-pass filtered at 30 Hz, and baseline was corrected relative to the 0.3 sec interval prior to the TS-onset. We used two single equivalent current dipoles (one for each hemisphere) to estimate the N1m source locations and orientations. Since previous MEG studies [22], [23], [24] reported that simultaneously presented BENs did not systematically influence the N1m source estimation, the MEG waveforms in the no-BEN condition, exhibiting the highest signal-to-noise ratio, were used for source estimation. The no-BEN condition was solely used for the determination of the N1m dipole source locations and orientations, in order to avoid circular data analysis [43]. A 0.01 sec time window around the N1m peak latency was used for dipole source estimation. The estimated source for each hemisphere of each subject was fixed in its location and orientation as a spatial filter [44], and the corresponding source strength waveforms were calculated for each BEN condition (BEN_1/4CB, BEN_1/2CB and BEN_1CB) and each session (distracted listening and focused listening).

### Statistical Analysis

In order to minimize the inter-individual and inter-session differences (i.e., head position, brain anatomy, head size, etc.), the N1m source strengths were normalized with respect to the N1m source strength averaged across the BEN_1/4CB, BEN_1/2CB and BEN_1CB conditions for each subject and hemisphere separately for each session. Thereafter, the N1m source strength ratios in the distracted and focused listening conditions were separately analyzed via repeated-measures ANOVA using the two factors BEN-TYPE (BEN_1/4CB, BEN_1/2CB, BEN_1CB) and GROUP (exposed, control). The behavioral responses (reaction time and error rate) in each BEN condition in focused listening were evaluated by repeated-measures ANOVA using BEN-TYPE (BEN_1/4CB, BEN_1/2CB, BEN_1CB, no BEN) and GROUP (exposed, control) as factors.
